# Ensuring the quality of multiple-choice exams administered to small cohorts: A cautionary tale

**DOI:** 10.1007/s40037-016-0322-0

**Published:** 2017-01-03

**Authors:** Meredith Young, Beth-Ann Cummings, Christina St-Onge

**Affiliations:** 1 Department of Medicine, McGill University, Montreal, Quebec Canada; 2 Centre for Medical Education, McGill University, Montreal, Quebec Canada; 3 Department of Medicine, Université de Sherbrooke, Sherbrooke, Quebec Canada; 4 Centre de Pédagogie des Sciences de la Santé, Université de Sherbrooke, Sherbrooke, Quebec Canada

**Keywords:** Item properties, Assessment, Multiple-choice examination

## Abstract

**Introduction:**

Multiple-choice questions (MCQs) are a cornerstone of assessment in medical education. Monitoring item properties (difficulty and discrimination) are important means of investigating examination quality. However, most item property guidelines were developed for use on large cohorts of examinees; little empirical work has investigated the suitability of applying guidelines to item difficulty and discrimination coefficients estimated for small cohorts, such as those in medical education. We investigated the extent to which item properties vary across multiple clerkship cohorts to better understand the appropriateness of using such guidelines with small cohorts.

**Methods:**

Exam results for 32 items from an MCQ exam were used. Item discrimination and difficulty coefficients were calculated for 22 cohorts (*n* = 10–15 students). Discrimination coefficients were categorized according to Ebel and Frisbie (1991). Difficulty coefficients were categorized according to three guidelines by Laveault and Grégoire (2014). Descriptive analyses examined variance in item properties across cohorts.

**Results:**

A large amount of variance in item properties was found across cohorts. Discrimination coefficients for items varied greatly across cohorts, with 29/32 (91%) of items occurring in both Ebel and Frisbie’s ‘poor’ and ‘excellent’ categories and 19/32 (59%) of items occurring in all five categories. For item difficulty coefficients, the application of different guidelines resulted in large variations in examination length (number of items removed ranged from 0 to 22).

**Discussion:**

While the psychometric properties of items can provide information on item and exam quality, they vary greatly in small cohorts. The application of guidelines with small exam cohorts should be approached with caution.

## What this paper adds

Item property estimates are often used to judge the quality of multiple-choice questions. Large amounts of variance are observed in estimates of difficulty and discrimination for items on multiple choice examinations used in small cohorts. Large amounts of variance in item property estimates suggest that conclusions based on these estimates should be drawn with caution and that decisions regarding item quality should be informed by many factors, including item property estimates, content representativeness, purpose of assessment, length of assessment, in consideration of cohort size to ensure decisions affecting final scores are done in the most defensible way possible.

## Introduction

Assessment serves several key functions in medical education: it is a gatekeeper, a feedback mechanism, a means to support learning [1–4], and a stepping stone in assuring competent practice (e. g., [5–7]). While programmes of assessment often include multiple assessment methods [8, 9], one of the most commonly used methods is the written exam based on multiple choice questions (MCQs). MCQs are known for their objectivity, ease of scoring, and wide sampling of broad content areas [10, 11]. MCQs are a pervasive item format in medical education, often appearing in national-level examinations (e. g. United States Medical Licensing Exam (USMLE) and Medical Council of Canada Qualifying Exam (MCCQE)).

While ubiquitous, multiple-choice based examinations require careful monitoring to ensure not only continued item and examination quality, but also a credible final score on which to base education judgments – including decisions regarding gate-keeping and remediation. One means of monitoring is to rely on item statistics or item properties, such as difficulty and discrimination coefficients. These item properties can be derived after exam administration, and thus are available to help administrators judge the quality of individual items and make decisions regarding the composition of the final examination score [12]. Pragmatically, if an item’s properties do not meet predetermined standards, the item may be excluded from the final score to derive a more appropriate final score, and then re-evaluated for later use (either maintained or removed from an ‘item bank’).

While alternate means of assessing item quality are available, one of the more commonly applied approaches in health professions education is that of Classical Test Theory (CTT). Within this framework, difficulty and discrimination coefficients are amongst the most commonly used metrics that examination administrators rely on to assist in quality assurance decisions. Standards for judging the appropriateness or quality of an MCQ can be found in the educational psychology literature [13–17] and have been adopted within health professions education to assist quality decisions. However, most item analysis guidelines were developed from, and intended for use in, examinations administered to large cohorts (such as national-level examinations) where item properties such as item difficulty and discrimination coefficients are taken to be stable (i. e. they do not vary between cohorts due to large examination cohort sizes) [12, 18–21]. Several guidelines exist for interpreting item difficulty and discrimination estimates [13–17]; however the cohort size needed to most appropriately apply item analysis guidelines varies from 30 to 200 examines[12, 18–21] with a general rule of thumb to have 5 to 10 times as many examinees as items [21]. Our collective experience suggests that item guidelines are frequently used in medical education cohorts that are smaller, particularly at the clerkship level.

Course-level leaders rarely have the opportunity to pursue formalized training in measurement, but are often responsible for overseeing the quality of the examinations used within their educational portfolios. In order to facilitate these roles, course leaders are often encouraged to attend local or national courses where psychometrics and approaches to examination quality are taught in brief sessions (e. g., [22]), and where they may be provided with easy-to-digest articles and guidelines that cite original measurement works (e. g., [23]). The educational opportunities intended for course leaders introduce concepts relating to the utility of item properties for quality monitoring but given the breadth of topics covered and short timelines, there is rarely an opportunity to discuss the stability or instability of item properties across different cohort sizes – discussions often present in other educational and measurement fields [12, 19, 20, 24]. Further, while universities often provide reports including information on item properties (for cohorts of all sizes), they are rarely accompanied by information on how to interpret these properties or how best to apply quality monitoring guidelines to the data presented.

The application of item analysis guidelines assumes that item properties are relatively stable, meaning that the estimates of difficulty and discrimination would change little between exam administrations. For example, according to this assumption a ‘difficult’ item remains difficult across different groups of similarly skilled examinees, which resonates well with our intuition and our intent when creating questions [12, 21]. However, if large amounts of item property variance are present in small cohorts, this would undermine the decisions reached while relying on these guidelines, such as choosing to retain an item or remove it from a total exam score or an item bank. Further, if item property of variance is observed, and this variance leads to different items being removed from exams, this may result in changes to the content and composition of an exam across cohorts of students. Large amounts of variance in item properties across cohorts of examinees would, therefore, challenge the appropriateness of applying these guidelines, calling into question the practice of applying item guidelines to small cohorts within a medical education context. As a first step in understanding whether we can use item analysis guidelines to inform our decision processes in small assessment cohorts, this study investigated the amount of variance observed in item properties when an MCQ examination was administered to several sequential clerkship cohorts.

## Method

A descriptive exploratory study was conducted. This study was approved by the McGill University Research Ethics Board (A10-E82-13A).

### Data collection

Exam results for a locally developed knowledge-based MCQ exam at the fourth year clerkship level were used. The exam was administered within the assessment programme of a senior clerkship rotation. This topic-based knowledge exam represented 40% of the final score for that clerkship rotation (the other 60% of total score derived from clinical assessment). The purpose of the exam was to assess students’ application of knowledge within the focal clerkship. The exam pass score was set at 60%, as is the institutional standard for the site of this study. All students participated in the same academic half-day teaching content, and all had the same set of recommended readings.

The questions within the exam were created, vetted, and adapted by the undergraduate education committee for the relevant clerkship, and were mapped to overall curricular and specific course objectives. Members of the undergraduate education committee for this clerkship were aware of item-writing guidelines; however, no formal quality assessment process for items (beyond peer-review, editing, and vetting) was done. And all represented single best answer questions (5 response options). The exam items were chosen from the item bank by the course director to represent a range of topic areas. Item statistics (i. e. difficulty and discrimination) were not deliberately used in the selection of examination items. The exam, comprising 50 items from a pool of 70 items, was administered to 22 cohorts of clerkship students (*n* = 10–15 students) between 2010 and 2013. Two cohorts between 2010 and 2013 were not included in the analysis as they contained only one individual, so calculation of discrimination coefficients was not possible.

### Dataset

Of the 70 banked MCQ items, 32 items occurred on all examinations. For each cohort of examinees, the difficulty coefficients (i. e. the proportion of candidates who answered the item correctly) and discrimination coefficients (the ‘corrected’ point-biserial correlation, i. e., the correlation between the item score and overall score (minus the item score)) were calculated for the 32 repeated examination items. Therefore, each of the 32 MCQ items included in this analysis had a total of 22 difficulty coefficients and 22 discrimination coefficients calculated from cohorts ranging in size from 10–15 students.

### Procedure

#### Classification of discrimination coefficients

Each discrimination coefficient (32 MCQ items with 22 coefficients each) was classified according to Ebel and Frisbie’s [13] guidelines for item quality, that is, coefficients <0.10 = poor discrimination; 0.10–0.19 = low discrimination; 0.20–0.29 = acceptable discrimination; 0.30–0.39 = good discrimination; and >0.40 = excellent discrimination.

#### Classification of difficulty coefficients

Difficulty coefficients were examined according to three guidelines proposed by Laveault and Grégoire [15] for item quality. For each item, the outcome of categorization was whether or not it should be excluded from the final examination score. According to guideline 1, an item should be excluded from the examination if the item difficulty was ± two standard deviations from the average difficulty of the examination. According to guideline 2, an item should be excluded if it fell ± two standard deviations from the passing score (here, set at 60%). According to guideline 3, an item should be excluded if difficulty was less than 0.2 or more than 0.8 (a common ‘rule of thumb’ for interpreting difficulty estimates). For each examination cohort, the number of items recommended to be excluded from the final score according to each guideline was recorded.

### Analysis

#### Identification of variance in item characteristics

The quantification of variance for difficulty and discrimination coefficients for the 32 MCQ items used in 22 repeated small cohorts was primarily descriptive. Using graphical representation, the discrimination and difficulty coefficients were plotted using a box and whisker plot. The discrimination and difficulty coefficients for a sample item were plotted in order to demonstrate the variance in item characteristics across repeated uses within a single item.

#### Outcome variance of item characteristics on exam composition

The exploration of the impact of variance in item properties on exam composition was primarily descriptive. For item discrimination, frequency analyses were conducted to capture the variance in coefficients (i. e., the number of times an item was in each of Ebel and Frisbie’s five categories) [13]. For difficulty coefficients, descriptive frequency analyses were conducted to capture the impact of applying difficulty guidelines as interpreted from Laveault and Grégoire [25] on exam length (number of items to be excluded from the total score).

## Results

### Identification of variance in item characteristics

**Fig. 1:**
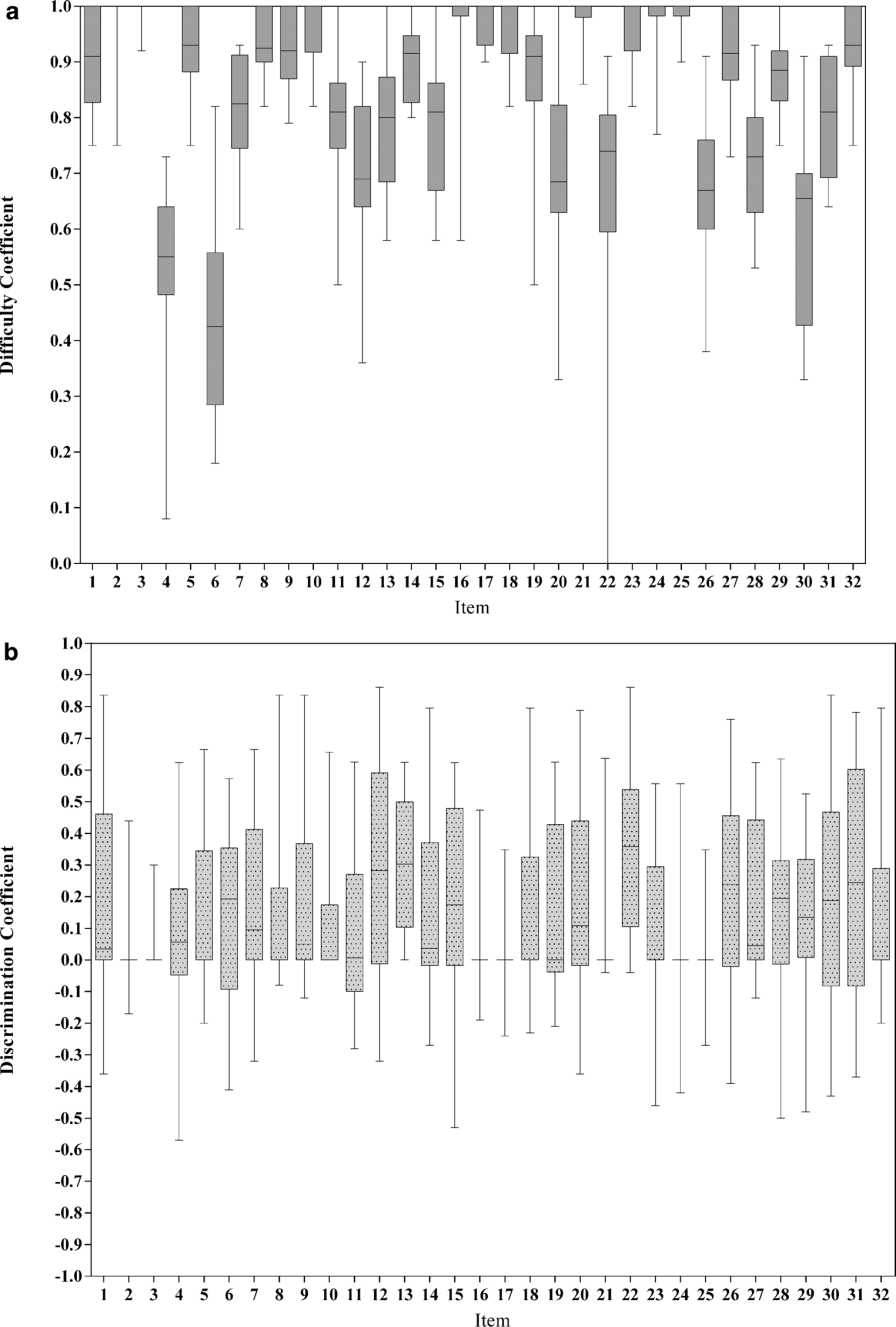
Variance in difficulty coefficients (*Panel* *A*) and discrimination coefficients (*Panel* *B*) across repeat use of 32 MCQ items across 22 small student clerkship cohorts (*n* = 10–15 students). Error bars represent range

The range of discrimination and difficulty coefficients for each item are illustrated in Fig. 1 (Panel A and B respectively). The majority of examination items display a large amount of variance in item property coefficients.

**Fig. 2:**
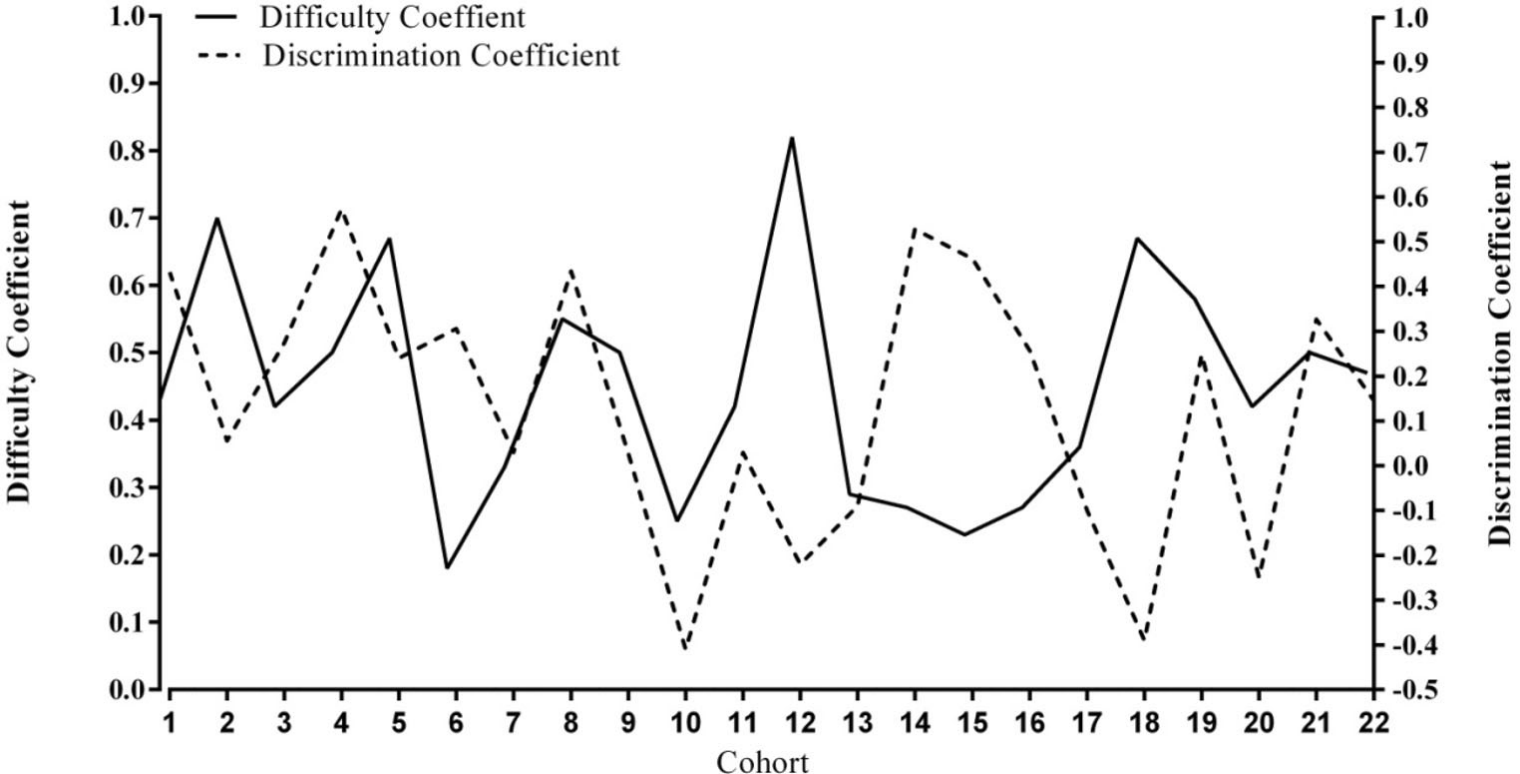
Variability in difficulty and discrimination indices for a single MCQ item (Item 6) graphed across cohort

While Fig. 1 shows the total amount of variance within and across items included in this study, it is difficult to visualize how this variation in item properties is reflected in individual examination cohorts. In order to assist with visualizing the variance of difficulty and discrimination indices for one sample item across cohort (time), a single MCQ item was graphed across cohorts in Fig. 2.

### Outcome variance of item characteristics on exam composition

#### Item discrimination

Discrimination coefficients for each individual item varied greatly across cohorts. Discrimination for 29 of 32 exam items (91% of MCQ items) was classified as both ‘poor’ and ‘excellent’ at least once. Nineteen of 32 (59%) items had discrimination coefficients in all five of Ebel and Frisbie’s [13] categories of discrimination (from poor to excellent). Details of the distribution of Ebel and Frisbie’s categorizations for each item can be found in Table 1.

**Table 1:** Frequency of items being categorized in each of Ebel and Frisbie’s [13] categories, displayed for each item

	Discrimination categorization				
Item	Poor	Low	Acceptable	Good	Excellent
1	12	0	2	2	6
2	19	1	0	0	2
3	20	1	0	1	0
4	12	4	3	2	1
5	14	0	0	5	3
6	10	1	4	2	5
7	11	2	1	2	6
8	14	2	2	2	2
9	12	1	3	3	3
10	16	3	1	1	1
11	14	1	3	1	3
12	7	1	4	2	8
13	5	4	2	5	6
14	12	2	2	1	5
15	8	4	2	1	7
16	19	0	1	0	2
17	18	1	1	2	0
18	15	0	2	0	5
19	13	3	0	0	6
20	11	3	1	1	6
21	19	0	0	1	2
22	5	1	3	3	10
23	16	0	1	2	3
24	19	0	1	1	1
25	20	0	1	1	0
26	9	2	0	5	6
27	12	1	1	1	7
28	8	3	5	2	4
29	9	6	2	2	3
30	9	3	1	1	8
31	9	1	2	2	8
32	13	2	2	1	4

#### Item difficulty

Mean exam difficulty (and associated standard deviation of item difficulty) was calculated independently for each cohort in order to apply guidelines 1 and 2. Mean difficulty ranged across cohorts from 0.80 to 0.89, and standard deviations ranged from 0.15 to 0.21. Decisions regarding whether to include or exclude an item from the final score varied widely by examination cohort and by guideline applied. Guideline 1 (exclude item if ±2 standard deviations from the average difficulty) resulted in a range of 0 to 17 items removed across cohorts. Guideline 2 (±2 standard deviations from the passing score) resulted in a range of 1 to 18 items removed across cohorts. Guideline 3 (remove any item below 0.2 or over 0.8) resulted in a range of 1 to 22 items removed across cohorts. Table 2 shows the frequency in which each item would be recommended to be removed (across cohorts) under each guideline proposed by Laveault and Grégoire [25].

**Table 2:** Frequency of items either removed or kept for the total exam score, presented by item, and for each quality monitoring guideline^a^

Item	Number of times item is removed from the total score applying Guideline 1	Number of times item is removed from the total score applying Guideline 2	Number of times item is removed from the total score applying Guideline 3
1	0	7	18
2	0	18	21
3	0	17	22
4	9	1	1
5	0	11	21
6	17	3	2
7	0	1	14
8	0	12	22
9	0	10	21
10	0	14	22
11	0	3	11
12	2	0	7
13	0	2	10
14	0	6	20
15	0	1	11
16	0	15	21
17	0	15	22
18	0	14	22
19	1	6	18
20	2	1	6
21	0	15	22
22	2	1	6
23	0	14	22
24	0	17	21
25	0	15	22
26	3	1	3
27	0	8	19
28	1	1	4
29	0	4	20
30	6	1	2
31	0	1	11
32	0	12	21

^a^Guideline 1: an item should be excluded from the examination if the item difficulty was ± two standard deviations from the average difficulty of the examination. Guideline 2: an item should be excluded if it fell ± two standard deviations from the passing score. Guideline 3: an item should be excluded if difficulty was less than 0.2 or more than 0.8 [15].

## Discussion

This study documented large amounts of variance in item difficulty and discrimination coefficients in multiple choice items repeatedly used in small cohorts of learners. Almost every item included in this study was categorized as having ‘excellent’ and ‘poor’ item discrimination, and over half of the items occurred in each of Ebel and Frisbie’s five categories of discrimination quality [13]. Large amounts of variance were also found for item difficulty, with substantial differences across cohorts in the likelihood of an item being recommended to be removed from or included in a final score.

For large cohorts of examination takers, item discrimination and item difficulty are assumed to be stable coefficients, properties that are inherent to the item [12, 19, 20, 24]. Consequently, removing poor quality items is thought to implicitly improve the quality of the examination. In the absence of evidence to the contrary, and with a paucity of reasonable alternatives to assure examination quality, our collective experience suggests that exam administrators are apt to use item analysis guidelines to inform decisions regarding whether or not to include an item in a total score, even for examinations administered to small cohorts of test-takers. However, in this study, a large amount of variance was observed for difficulty and discrimination coefficients across cohorts. While this study examined the instability of these estimates across multiple cohorts, administrators are often faced with the task of assuring examination quality with little or no historic data regarding item performance; there is often a pragmatic need to decide whether or not an item should be removed from a total score despite having data from only one cohort, or one time-point. This study demonstrates the instability of difficulty and discrimination coefficients, which may call into question the application of item analysis guidelines for assessment data generated by small cohorts. This large amount of variance in item properties may not come as a surprise to those familiar with the development of item quality guidelines; however, given the use of these guidelines with the small cohorts common in medical education, we believe that it is imperative to illustrate the variance in item properties across small cohorts within this context.

For individuals overseeing examinations administered to small cohorts, such as clerkship examinations, the application of item analysis guidelines in the context of small cohorts may actually undermine the intended goals of quality assessment across small cohorts. For example, in an attempt to ensure equal difficulty of exams across cohorts, a single exam might be used across time and training location (supposing it to be equivalent across contexts and academic years). Similarly, a ‘core’ set of items may be used across exam administrations, sampling similar content across students in an attempt to ensure equivalence and comparability of performance, or assessment of critical knowledge. However, if the same item, used across time, generates vastly different item properties across cohorts, the likelihood of keeping or removing a given item from an exam score will also vary. The variation in whether or not an item is included in a final score could consequently impact content representativeness and overall exam difficulty. These components of ‘good assessment’ are of utmost importance to curriculum developers, course directors, and educational leaders, due to the accreditation requirement for equivalent and high quality assessments over time.

This study has some limitations. It relied on archived examination reports, and so only discrimination and difficulty coefficients were available, not raw examination performance for individuals. While this approach ensures the anonymity of examinees, it makes it impossible to examine the consequences of the application of item analysis guidelines in small cohorts on examination reliability and overall difficulty. Further, the purpose of this study was to explicitly document the presence of variance in item properties in small cohorts of examinees in health professions education, and as such we are currently unable to provide guidance regarding the boundaries of when, how, and under what circumstances item analysis guidelines can be applied without negative consequences to examination reliability, length, or difficulty, or how various approaches to equivalence may remedy this. This study also applied item analysis guidelines in their ‘purest’ form; discrimination and difficulty were considered individually, and without information regarding exam blueprint or content coverage. This may represent an under-nuanced application of item analysis guidelines, but we have few details regarding how item analysis guidelines are currently used or contextualized within health professions education. To our knowledge no formal evaluation of quality for individual items was done beyond peer-review, revision and vetting, representing a potential limitation to our study. We have no current access to item writers, nor to formal evaluations of item quality or data supporting construct alignment. Items were generated to align with overall curricular and course-specific learning objectives, and clinical clerks participated in the same academic half-days. It is possible that other factors such as varying clinical teacher quality, specific metrics of item quality, or undetected issues of construct alignment may contribute to item property variance. While we are currently unable to parse out the relative contribution of these factors to the item property instability we observed within this study, the instability of estimates of parameters in small cohorts is well supported in other domains (e. g. Law of large numbers in statistics).

The purpose of this study was to document and explore the extent of variability in item characteristics in MCQ exams given to small cohorts; we can provide few recommendations beyond cautioning the use of item analysis guidelines with small cohorts. Within the limits of the current work, we would suggest that recommendations based on guidelines (to remove an item from a total score or not) should be considered in parallel with other factors such as content representativeness, and that it is good practice to consult individuals with measurement expertise when making quality monitoring decisions for assessment, particularly in the context of assessment data derived from small cohorts. Future work will hopefully be able to provide stronger guidance and recommendations on the appropriate, or at least harm-minimizing, contexts for appropriate use of item analysis guidelines.

Item properties are one means of examining item and examination quality, and often underlie important assessment decisions such as whether to exclude items from the final score. Variations due to small cohorts of exam takers raise concerns for assessment decisions based on these metrics, and the application of item analysis guidelines should be approached with caution within small assessment cohorts.
